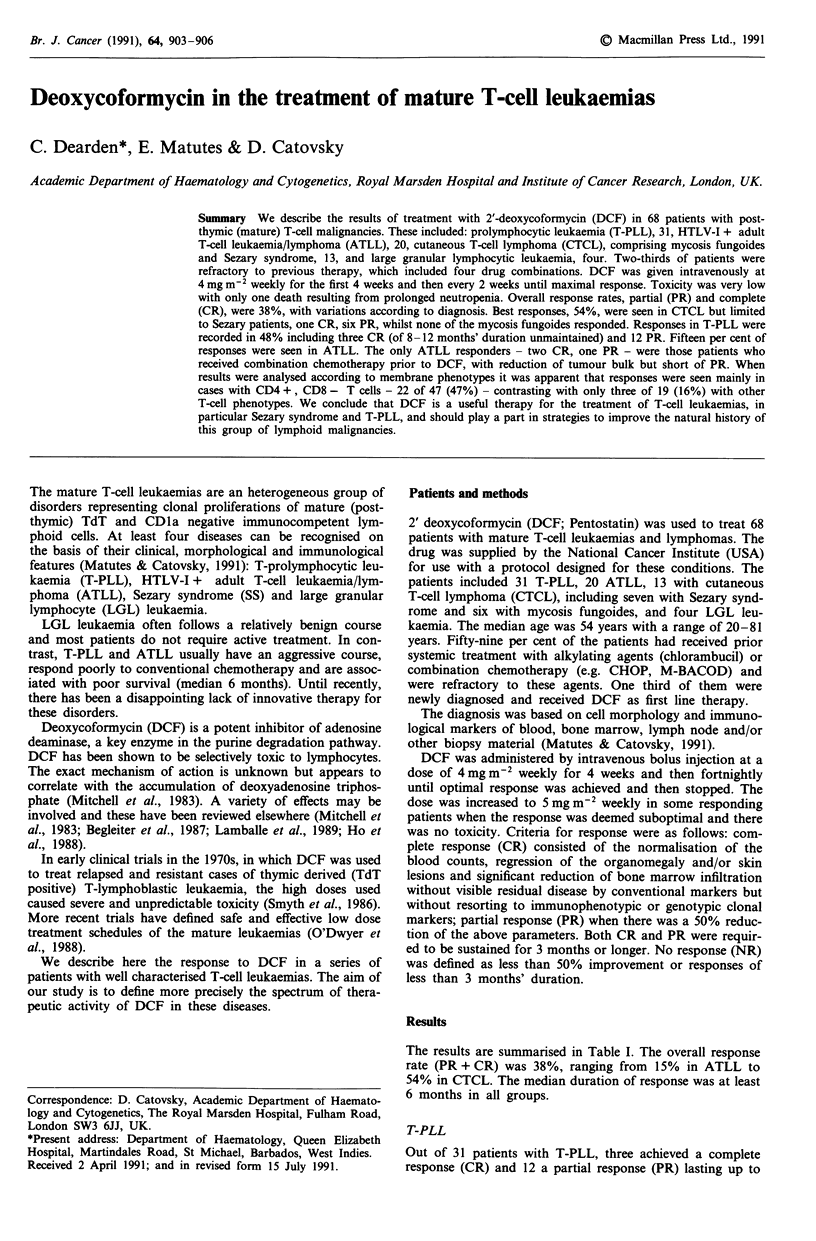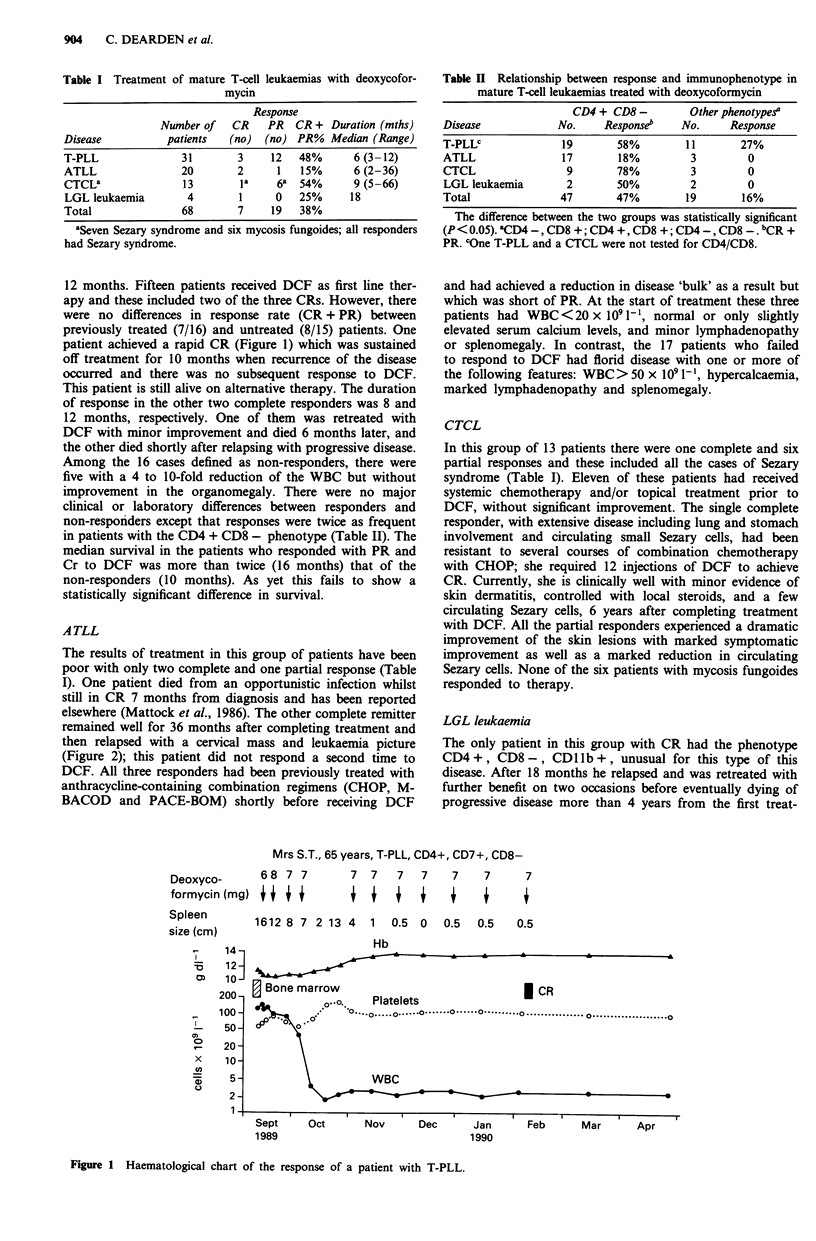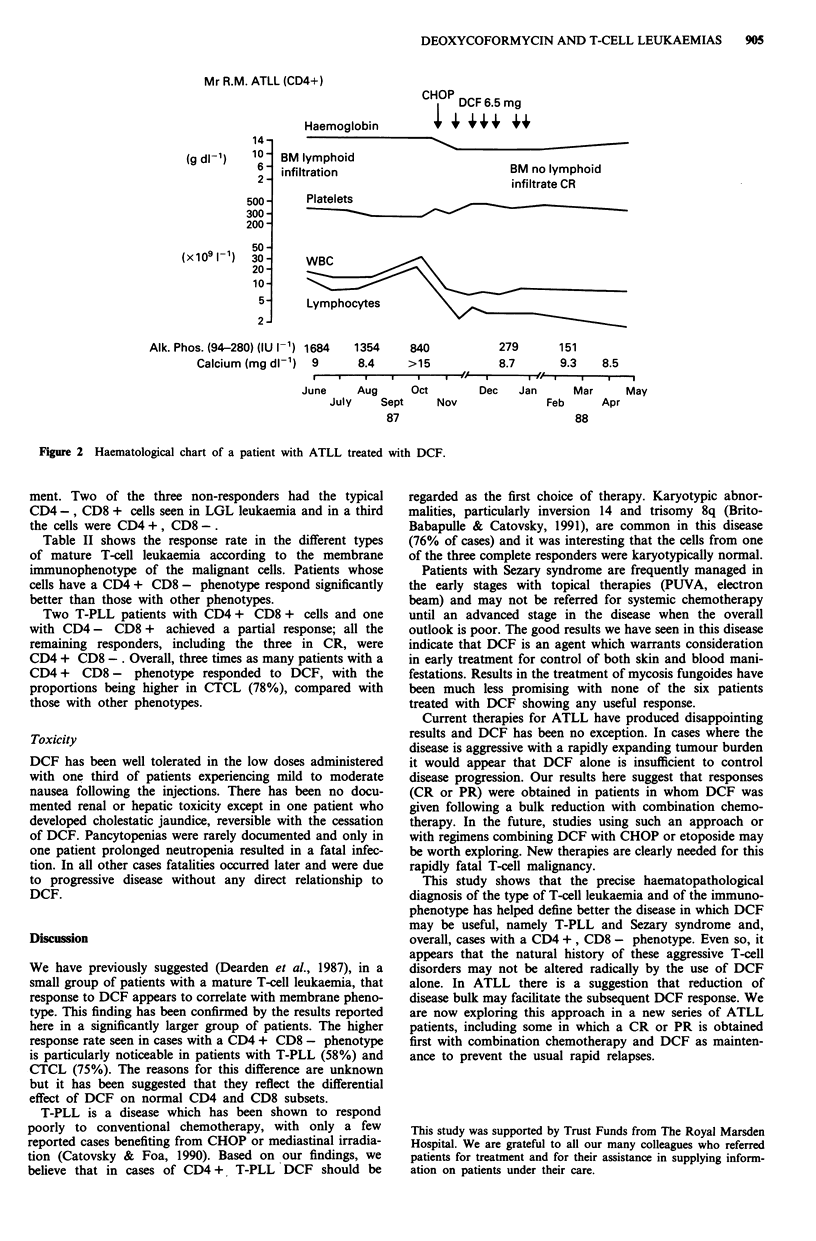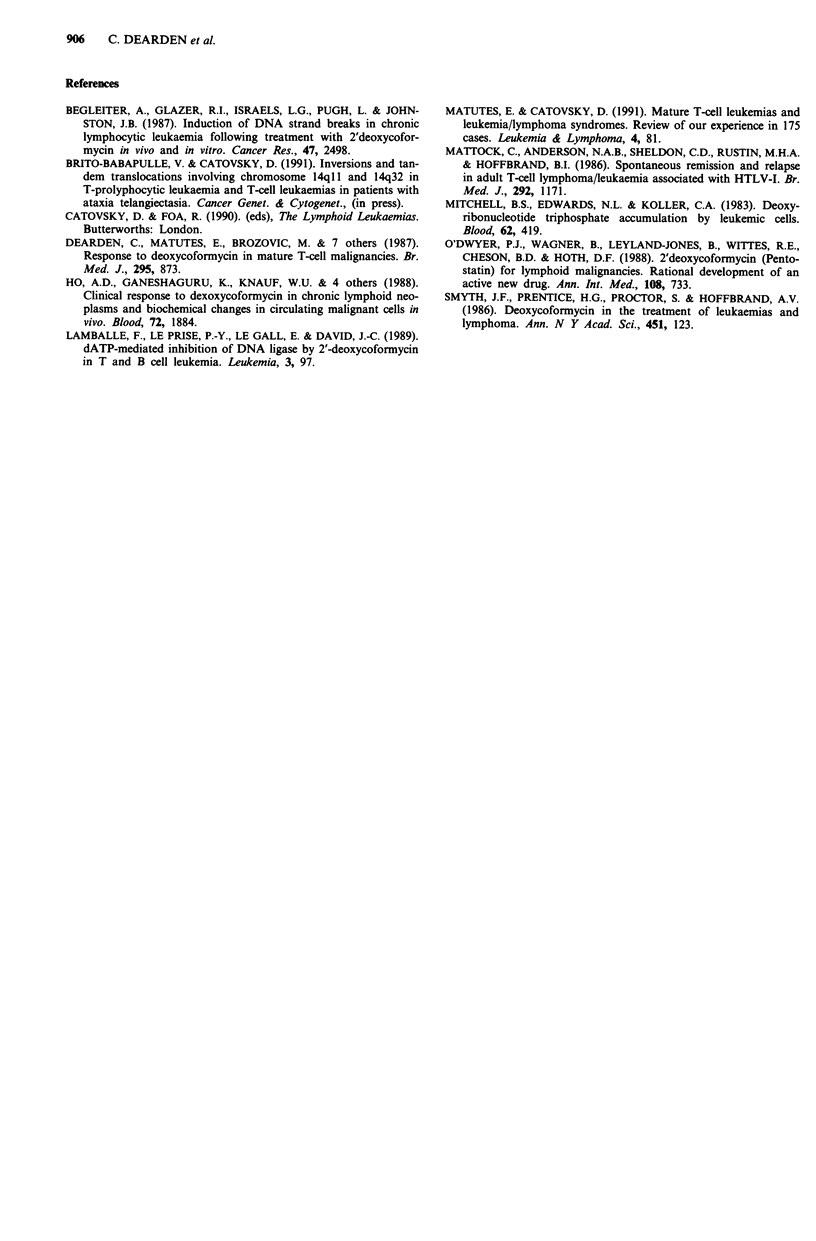# Deoxycoformycin in the treatment of mature T-cell leukaemias.

**DOI:** 10.1038/bjc.1991.423

**Published:** 1991-11

**Authors:** C. Dearden, E. Matutes, D. Catovsky

**Affiliations:** Academic Department of Haematology and Cytogenetics, Royal Marsden Hospital, London, UK.

## Abstract

We describe the results of treatment with 2'-deoxycoformycin (DCF) in 68 patients with post-thymic (mature) T-cell malignancies. These included: prolymphocytic leukaemia (T-PLL), 31, HTLV-1 + adult T-cell leukaemia/lymphoma (ATLL), 20, cutaneous T-cell lymphoma (CTCL), comprising mycosis fungoides and Sezary syndrome, 13, and large granular lymphocytic leukaemia, four. Two-thirds of patients were refractory to previous therapy, which included four drug combinations. DCF was given intravenously at 4 mg m-2 weekly for the first 4 weeks and then every 2 weeks until maximal response. Toxicity was very low with only one death resulting from prolonged neutropenia. Overall response rates, partial (PR) and complete. (CR), were 38%, with variations according to diagnosis. Best responses, 54%, were seen in CTCL but limited to Sezary patients, one CR, six PR, whilst none of the mycosis fungoides responded. Responses in T-PLL were recorded in 48% including three CR (of 8-12 months' duration unmaintained) and 12 PR. Fifteen per cent of responses were seen in ATLL. The only ATLL responders - two CR, one PR - were those patients who received combination chemotherapy prior to DCF, with reduction of tumour bulk but short of PR. When results were analysed according to membrane phenotypes it was apparent that responses were seen mainly in cases with CD4+, CD8- T cells -22 of 47 (47%) - contrasting with only three of 19 (16%) with other T-cell phenotypes. We conclude that DCF is a useful therapy for the treatment of T-cell leukaemias, in particular Sezary syndrome and T-PLL, and should play a part in strategies to improve the natural history of this group of lymphoid malignancies.


Br. J. Cancer (1991), 64, 903-906 © Macmillan Press Ltd., 1991

### Deoxycoformycin in the treatment of mature T-cell leukaemias

C. Dearden*, E. Matutes & D. Catovsky

*Academic Department of Haematology and Cytogenetics, Royal Marsden Hospital and Institute of Cancer Research, London, UK.*

**Summary** We describe the results of treatment with 2'-deoxycoformycin (DCF) in 68 patients with post-thymic (mature) T-cell malignancies. These included: prolymphocytic leukaemia (T-PLL), 31, HTLV-I + adult T-cell leukaemia/lymphoma (ATLL), 20, cutaneous T-cell lymphoma (CTCL), comprising mycosis fungoides and Sezary syndrome, 13, and large granular lymphocytic leukaemia, four. Two-thirds of patients were refractory to previous therapy, which included four drug combinations. DCF was given intravenously at $4 \text{ mg m}^{-2}$ weekly for the first 4 weeks and then every 2 weeks until maximal response. Toxicity was very low with only one death resulting from prolonged neutropenia. Overall response rates, partial (PR) and complete (CR), were 38%, with variations according to diagnosis. Best responses, 54%, were seen in CTCL but limited to Sezary patients, one CR, six PR, whilst none of the mycosis fungoides responded. Responses in T-PLL were recorded in 48% including three CR (of 8-12 months' duration unmaintained) and 12 PR. Fifteen per cent of responses were seen in ATLL. The only ATLL responders - two CR, one PR - were those patients who received combination chemotherapy prior to DCF, with reduction of tumour bulk but short of PR. When results were analysed according to membrane phenotypes it was apparent that responses were seen mainly in cases with CD4 +, CD8 - T cells - 22 of 47 (47%) - contrasting with only three of 19 (16%) with other T-cell phenotypes. We conclude that DCF is a useful therapy for the treatment of T-cell leukaemias, in particular Sezary syndrome and T-PLL, and should play a part in strategies to improve the natural history of this group of lymphoid malignancies.

The mature T-cell leukaemias are an heterogeneous group of disorders representing clonal proliferations of mature (post-thymic) TdT and CD1a negative immunocompetent lymphoid cells. At least four diseases can be recognised on the basis of their clinical, morphological and immunological features (Matutes & Catovsky, 1991): T-prolymphocytic leukaemia (T-PLL), HTLV-I + adult T-cell leukaemia/lymphoma (ATLL), Sezary syndrome (SS) and large granular lymphocyte (LGL) leukaemia.

LGL leukaemia often follows a relatively benign course and most patients do not require active treatment. In contrast, T-PLL and ATLL usually have an aggressive course, respond poorly to conventional chemotherapy and are associated with poor survival (median 6 months). Until recently, there has been a disappointing lack of innovative therapy for these disorders.

Deoxycoformycin (DCF) is a potent inhibitor of adenosine deaminase, a key enzyme in the purine degradation pathway. DCF has been shown to be selectively toxic to lymphocytes. The exact mechanism of action is unknown but appears to correlate with the accumulation of deoxyadenosine triphosphate (Mitchell *et al.*, 1983). A variety of effects may be involved and these have been reviewed elsewhere (Mitchell *et al.*, 1983; Begleiter *et al.*, 1987; Lamballe *et al.*, 1989; Ho *et al.*, 1988).

In early clinical trials in the 1970s, in which DCF was used to treat relapsed and resistant cases of thymic derived (TdT positive) T-lymphoblastic leukaemia, the high doses used caused severe and unpredictable toxicity (Smyth *et al.*, 1986). More recent trials have defined safe and effective low dose treatment schedules of the mature leukaemias (O'Dwyer *et al.*, 1988).

We describe here the response to DCF in a series of patients with well characterised T-cell leukaemias. The aim of our study is to define more precisely the spectrum of therapeutic activity of DCF in these diseases.

Correspondence: D. Catovsky, Academic Department of Haematology and Cytogenetics, The Royal Marsden Hospital, Fulham Road, London SW3 6JJ, UK.
*Present address: Department of Haematology, Queen Elizabeth Hospital, Martindales Road, St Michael, Barbados, West Indies.
Received 2 April 1991; and in revised form 15 July 1991.

### Patients and methods

2' deoxycoformycin (DCF; Pentostatin) was used to treat 68 patients with mature T-cell leukaemias and lymphomas. The drug was supplied by the National Cancer Institute (USA) for use with a protocol designed for these conditions. The patients included 31 T-PLL, 20 ATLL, 13 with cutaneous T-cell lymphoma (CTCL), including seven with Sezary syndrome and six with mycosis fungoides, and four LGL leukaemia. The median age was 54 years with a range of 20-81 years. Fifty-nine per cent of the patients had received prior systemic treatment with alkylating agents (chlorambucil) or combination chemotherapy (e.g. CHOP, M-BACOD) and were refractory to these agents. One third of them were newly diagnosed and received DCF as first line therapy.

The diagnosis was based on cell morphology and immunological markers of blood, bone marrow, lymph node and/or other biopsy material (Matutes & Catovsky, 1991).

DCF was administered by intravenous bolus injection at a dose of $4 \text{ mg m}^{-2}$ weekly for 4 weeks and then fortnightly until optimal response was achieved and then stopped. The dose was increased to $5 \text{ mg m}^{-2}$ weekly in some responding patients when the response was deemed suboptimal and there was no toxicity. Criteria for response were as follows: complete response (CR) consisted of the normalisation of the blood counts, regression of the organomegaly and/or skin lesions and significant reduction of bone marrow infiltration without visible residual disease by conventional markers but without resorting to immunophenotypic or genotypic clonal markers; partial response (PR) when there was a 50% reduction of the above parameters. Both CR and PR were required to be sustained for 3 months or longer. No response (NR) was defined as less than 50% improvement or responses of less than 3 months' duration.

### Results

The results are summarised in Table I. The overall response rate (PR + CR) was 38%, ranging from 15% in ATLL to 54% in CTCL. The median duration of response was at least 6 months in all groups.

### *T-PLL*

Out of 31 patients with T-PLL, three achieved a complete response (CR) and 12 a partial response (PR) lasting up to

**Table I** Treatment of mature T-cell leukaemias with deoxycoformycin

| Disease | Number of patients | CR (no) | PR (no) | CR + PR% | Duration (mths) Median (Range) |
|---|---|---|---|---|---|
| T-PLL | 31 | 3 | 12 | 48% | 6 (3–12) |
| ATLL | 20 | 2 | 1 | 15% | 6 (2–36) |
| CTCL[a] | 13 | 1[a] | 6[a] | 54% | 9 (5–66) |
| LGL leukaemia | 4 | 1 | 0 | 25% | 18 |
| Total | 68 | 7 | 19 | 38% | |

[a]Seven Sezary syndrome and six mycosis fungoides; all responders had Sezary syndrome.

**Table II** Relationship between response and immunophenotype in mature T-cell leukaemias treated with deoxycoformycin

| Disease | CD4 + CD8 – No. | CD4 + CD8 – Response[b] | Other phenotypes[a] No. | Other phenotypes[a] Response |
|---|---|---|---|---|
| T-PLL[c] | 19 | 58% | 11 | 27% |
| ATLL | 17 | 18% | 3 | 0 |
| CTCL | 9 | 78% | 3 | 0 |
| LGL leukaemia | 2 | 50% | 2 | 0 |
| Total | 47 | 47% | 19 | 16% |

The difference between the two groups was statistically significant ($P<0.05$). [a]CD4 –, CD8 +; CD4 +, CD8 +; CD4 –, CD8 –. [b]CR + PR. [c]One T-PLL and a CTCL were not tested for CD4/CD8.

12 months. Fifteen patients received DCF as first line therapy and these included two of the three CRs. However, there were no differences in response rate (CR + PR) between previously treated (7/16) and untreated (8/15) patients. One patient achieved a rapid CR (Figure 1) which was sustained off treatment for 10 months when recurrence of the disease occurred and there was no subsequent response to DCF. This patient is still alive on alternative therapy. The duration of response in the other two complete responders was 8 and 12 months, respectively. One of them was retreated with DCF with minor improvement and died 6 months later, and the other died shortly after relapsing with progressive disease. Among the 16 cases defined as non-responders, there were five with a 4 to 10-fold reduction of the WBC but without improvement in the organomegaly. There were no major clinical or laboratory differences between responders and non-responders except that responses were twice as frequent in patients with the CD4 + CD8 – phenotype (Table II). The median survival in the patients who responded with PR and Cr to DCF was more than twice (16 months) that of the non-responders (10 months). As yet this fails to show a statistically significant difference in survival.

### *ATLL*

The results of treatment in this group of patients have been poor with only two complete and one partial response (Table I). One patient died from an opportunistic infection whilst still in CR 7 months from diagnosis and has been reported elsewhere (Mattock *et al.*, 1986). The other complete remitter remained well for 36 months after completing treatment and then relapsed with a cervical mass and leukaemia picture (Figure 2); this patient did not respond a second time to DCF. All three responders had been previously treated with anthracycline-containing combination regimens (CHOP, M-BACOD and PACE-BOM) shortly before receiving DCF and had achieved a reduction in disease 'bulk' as a result but which was short of PR. At the start of treatment these three patients had WBC $<20\times10^9\,l^{-1}$, normal or only slightly elevated serum calcium levels, and minor lymphadenopathy or splenomegaly. In contrast, the 17 patients who failed to respond to DCF had florid disease with one or more of the following features: WBC $>50\times10^9\,l^{-1}$, hypercalcaemia, marked lymphadenopathy and splenomegaly.

### *CTCL*

In this group of 13 patients there were one complete and six partial responses and these included all the cases of Sezary syndrome (Table I). Eleven of these patients had received systemic chemotherapy and/or topical treatment prior to DCF, without significant improvement. The single complete responder, with extensive disease including lung and stomach involvement and circulating small Sezary cells, had been resistant to several courses of combination chemotherapy with CHOP; she required 12 injections of DCF to achieve CR. Currently, she is clinically well with minor evidence of skin dermatitis, controlled with local steroids, and a few circulating Sezary cells, 6 years after completing treatment with DCF. All the partial responders experienced a dramatic improvement of the skin lesions with marked symptomatic improvement as well as a marked reduction in circulating Sezary cells. None of the six patients with mycosis fungoides responded to therapy.

### *LGL leukaemia*

The only patient in this group with CR had the phenotype CD4 +, CD8 –, CD11b +, unusual for this type of this disease. After 18 months he relapsed and was retreated with further benefit on two occasions before eventually dying of progressive disease more than 4 years from the first treat-

**Figure 1** Haematological chart of the response of a patient with T-PLL.

**Figure 2** Haematological chart of a patient with ATLL treated with DCF.

ment. Two of the three non-responders had the typical CD4 – , CD8 + cells seen in LGL leukaemia and in a third the cells were CD4 + , CD8 – .

Table II shows the response rate in the different types of mature T-cell leukaemia according to the membrane immunophenotype of the malignant cells. Patients whose cells have a CD4 + CD8 – phenotype respond significantly better than those with other phenotypes.

Two T-PLL patients with CD4 + CD8 + cells and one with CD4 – CD8 + achieved a partial response; all the remaining responders, including the three in CR, were CD4 + CD8 – . Overall, three times as many patients with a CD4 + CD8 – phenotype responded to DCF, with the proportions being higher in CTCL (78%), compared with those with other phenotypes.

*Toxicity*

DCF has been well tolerated in the low doses administered with one third of patients experiencing mild to moderate nausea following the injections. There has been no documented renal or hepatic toxicity except in one patient who developed cholestatic jaundice, reversible with the cessation of DCF. Pancytopenias were rarely documented and only in one patient prolonged neutropenia resulted in a fatal infection. In all other cases fatalities occurred later and were due to progressive disease without any direct relationship to DCF.

### Discussion

We have previously suggested (Dearden *et al.*, 1987), in a small group of patients with a mature T-cell leukaemia, that response to DCF appears to correlate with membrane phenotype. This finding has been confirmed by the results reported here in a significantly larger group of patients. The higher response rate seen in cases with a CD4 + CD8 – phenotype is particularly noticeable in patients with T-PLL (58%) and CTCL (75%). The reasons for this difference are unknown but it has been suggested that they reflect the differential effect of DCF on normal CD4 and CD8 subsets.

T-PLL is a disease which has been shown to respond poorly to conventional chemotherapy, with only a few reported cases benefiting from CHOP or mediastinal irradiation (Catovsky & Foa, 1990). Based on our findings, we believe that in cases of CD4 + T-PLL DCF should be regarded as the first choice of therapy. Karyotypic abnormalities, particularly inversion 14 and trisomy 8q (Brito-Babapulle & Catovsky, 1991), are common in this disease (76% of cases) and it was interesting that the cells from one of the three complete responders were karyotypically normal.

Patients with Sezary syndrome are frequently managed in the early stages with topical therapies (PUVA, electron beam) and may not be referred for systemic chemotherapy until an advanced stage in the disease when the overall outlook is poor. The good results we have seen in this disease indicate that DCF is an agent which warrants consideration in early treatment for control of both skin and blood manifestations. Results in the treatment of mycosis fungoides have been much less promising with none of the six patients treated with DCF showing any useful response.

Current therapies for ATLL have produced disappointing results and DCF has been no exception. In cases where the disease is aggressive with a rapidly expanding tumour burden it would appear that DCF alone is insufficient to control disease progression. Our results here suggest that responses (CR or PR) were obtained in patients in whom DCF was given following a bulk reduction with combination chemotherapy. In the future, studies using such an approach or with regimens combining DCF with CHOP or etoposide may be worth exploring. New therapies are clearly needed for this rapidly fatal T-cell malignancy.

This study shows that the precise haematopathological diagnosis of the type of T-cell leukaemia and of the immunophenotype has helped define better the disease in which DCF may be useful, namely T-PLL and Sezary syndrome and, overall, cases with a CD4 + , CD8 – phenotype. Even so, it appears that the natural history of these aggressive T-cell disorders may not be altered radically by the use of DCF alone. In ATLL there is a suggestion that reduction of disease bulk may facilitate the subsequent DCF response. We are now exploring this approach in a new series of ATLL patients, including some in which a CR or PR is obtained first with combination chemotherapy and DCF as maintenance to prevent the usual rapid relapses.

This study was supported by Trust Funds from The Royal Marsden Hospital. We are grateful to all our many colleagues who referred patients for treatment and for their assistance in supplying information on patients under their care.

### References

BEGLEITER, A., GLAZER, R.I., ISRAELS, L.G., PUGH, L. & JOHNSTON, J.B. (1987). Induction of DNA strand breaks in chronic lymphocytic leukaemia following treatment with 2'deoxycoformycin *in vivo* and *in vitro*. *Cancer Res.*, **47**, 2498.

BRITO-BABAPULLE, V. & CATOVSKY, D. (1991). Inversions and tandem translocations involving chromosome 14q11 and 14q32 in T-prolyphocytic leukaemia and T-cell leukaemias in patients with ataxia telangiectasia. *Cancer Genet. & Cytogenet.*, (in press).

CATOVSKY, D. & FOA, R. (1990). (eds), *The Lymphoid Leukaemias*. Butterworths: London.

DEARDEN, C., MATUTES, E., BROZOVIC, M. & 7 others (1987). Response to deoxycoformycin in mature T-cell malignancies. *Br. Med. J.*, **295**, 873.

HO, A.D., GANESHAGURU, K., KNAUF, W.U. & 4 others (1988). Clinical response to dexoxycoformycin in chronic lymphoid neoplasms and biochemical changes in circulating malignant cells *in vivo*. *Blood*, **72**, 1884.

LAMBALLE, F., LE PRISE, P.-Y., LE GALL, E. & DAVID, J.-C. (1989). dATP-mediated inhibition of DNA ligase by 2'-deoxycoformycin in T and B cell leukemia. *Leukemia*, **3**, 97.

MATUTES, E. & CATOVSKY, D. (1991). Mature T-cell leukemias and leukemia/lymphoma syndromes. Review of our experience in 175 cases. *Leukemia & Lymphoma*, **4**, 81.

MATTOCK, C., ANDERSON, N.A.B., SHELDON, C.D., RUSTIN, M.H.A. & HOFFBRAND, B.I. (1986). Spontaneous remission and relapse in adult T-cell lymphoma/leukaemia associated with HTLV-I. *Br. Med. J.*, **292**, 1171.

MITCHELL, B.S., EDWARDS, N.L. & KOLLER, C.A. (1983). Deoxyribonucleotide triphosphate accumulation by leukemic cells. *Blood*, **62**, 419.

O'DWYER, P.J., WAGNER, B., LEYLAND-JONES, B., WITTES, R.E., CHESON, B.D. & HOTH, D.F. (1988). 2'deoxycoformycin (Pentostatin) for lymphoid malignancies. Rational development of an active new drug. *Ann. Int. Med.*, **108**, 733.

SMYTH, J.F., PRENTICE, H.G., PROCTOR, S. & HOFFBRAND, A.V. (1986). Deoxycoformycin in the treatment of leukaemias and lymphoma. *Ann. N Y Acad. Sci.*, **451**, 123.